# Enhanced Diabetes Detection and Blood Glucose Prediction Using TinyML-Integrated E-Nose and Breath Analysis: A Novel Approach Combining Synthetic and Real-World Data

**DOI:** 10.3390/bioengineering11111065

**Published:** 2024-10-25

**Authors:** Alberto Gudiño-Ochoa, Julio Alberto García-Rodríguez, Jorge Ivan Cuevas-Chávez, Raquel Ochoa-Ornelas, Antonio Navarrete-Guzmán, Carlos Vidrios-Serrano, Daniel Alejandro Sánchez-Arias

**Affiliations:** 1 Electronics Department, Tecnológico Nacional de México/Instituto Tecnológico de Ciudad Guzmán, Ciudad Guzmán 49100, Mexico; m21290934@cdguzman.tecnm.mx (A.G.-O.); m22290910@cdguzman.tecnm.mx (J.I.C.-C.); m21290935@cdguzman.tecnm.mx (D.A.S.-A.); 2 Centro Universitario del Sur (CUSUR), Departamento de Ciencias Computacionales e Innovación Tecnológica, Universidad de Guadalajara, Ciudad Guzmán 49000, Mexico; 3 Systems and Computation Department, Tecnológico Nacional de México/Instituto Tecnológico de Ciudad Guzmán, Ciudad Guzmán 49100, Mexico; raquel.oo@cdguzman.tecnm.mx; 4 Electrical and Electronic Engineering Department, Tecnológico Nacional de México/Instituto Tecnológico de Tepic, Tepic 63175, Mexico; antonio.navarrete@uan.edu.mx; 5 Academic Unit of Basic Sciences and Engineering, Universidad Autónoma de Nayarit, Tepic 63000, Mexico; carlos.vidrios@uan.edu.mx

**Keywords:** electronic nose, blood glucose prediction, diabetes, TinyML, exhaled breath analysis, machine learning, sensor array

## Abstract

Diabetes mellitus, a chronic condition affecting millions worldwide, necessitates continuous monitoring of blood glucose level (BGL). The increasing prevalence of diabetes has driven the development of non-invasive methods, such as electronic noses (e-noses), for analyzing exhaled breath and detecting biomarkers in volatile organic compounds (VOCs). Effective machine learning models require extensive patient data to ensure accurate BGL predictions, but previous studies have been limited by small sample sizes. This study addresses this limitation by employing conditional generative adversarial networks (CTGAN) to generate synthetic data from real-world tests involving 29 healthy and 29 diabetic participants, resulting in over 14,000 new synthetic samples. These data were used to validate machine learning models for diabetes detection and BGL prediction, integrated into a Tiny Machine Learning (TinyML) e-nose system for real-time analysis. The proposed models achieved an 86% accuracy in BGL identification using LightGBM (Light Gradient Boosting Machine) and a 94.14% accuracy in diabetes detection using Random Forest. These results demonstrate the efficacy of enhancing machine learning models with both real and synthetic data, particularly in non-invasive systems integrating e-noses with TinyML. This study signifies a major advancement in non-invasive diabetes monitoring, underscoring the transformative potential of TinyML-powered e-nose systems in healthcare applications.

## 1. Introduction

The global prevalence of diabetes mellitus (DM) continues to rise, with projections estimating over 783 million cases by 2045, particularly in low- and middle-income countries [1,2]. Type 2 diabetes mellitus (T2DM) constitutes approximately 90% of all cases, while Type 1 diabetes mellitus (T1DM) and gestational diabetes mellitus (GDM) represent the remainder. These conditions are associated with severe complications such as nerve damage, cardiovascular issues, and an increased risk of premature death [3,4,5].

DM arises from the body’s inability to produce or properly utilize insulin, leading to imbalances in BGL. Regular monitoring of BGL is crucial for managing symptoms and enhancing the quality of life [6,7]. However, traditional invasive methods, such as glucose meters, though accurate, are associated with pain, discomfort, and risks related to blood-borne pathogen transmission, including hepatitis and human immunodeficiency virus (HIV) [8,9,10]. Although reliable, laboratory-based tests can be both costly and time-consuming [11,12].

Non-invasive methods for BGL prediction and DM detection, such as the analysis of tears, saliva, urine, and breath, have shown promise but face limitations in precision and sensitivity [12,13,14]. Additionally, advanced techniques like radiofrequency-based methods and optical methods, such as photoplethysmography (PPG), are costly and dependent on direct sampling [15].

Breath analysis, particularly with the integration of e-noses, has emerged as a highly promising approach. Exhaled breath contains VOCs, that serve as biomarkers, forming a unique ‘breathprint’. When these biomarkers interact with gas sensors in the e-nose, they convert chemical signals into electrical signals, reflecting the concentration of target gas molecules. Unlike advanced gas analysis techniques such as gas chromatography–mass spectrometry (GC/MS), selected ion flow tube mass spectrometry (SIFT-MS), and proton transfer reaction mass spectrometry (PTR-MS), which are more accurate but expensive and less portable, e-noses offer a cost-effective and portable alternative [12,13,14,16].

E-noses, enhanced with machine learning (ML) and deep learning (DL) models, have improved accuracy and speed in non-invasive diagnostics [13]. Several studies have applied e-noses for detecting metabolic disorders, including DM, by analyzing elevated acetone levels in breath, a biomarker for high BGL; the inability of cells to absorb glucose leads to an abnormal increase in ketone bodies, including acetone, a volatile compound exhaled by the body [13,14,17,18,19]. This approach offers a non-invasive, rapid diagnostic method for diabetes, showing significant promise for glucose monitoring and diabetes diagnosis.

Despite these advancements, many studies remain limited by small sample sizes and substantial variability among patients with T1DM, T2DM, and healthy controls. A larger, more diverse sample size would improve the robustness and accuracy of ML and DL models. Additionally, patient variability is significant, as pathological changes alter breath composition [12,13].

For example, Yan et al. achieved over 90% sensitivity and specificity for predicting BGL using sensor arrays, with a mean relative absolute error (MRAE) of 21.7% [20]. Lekha and Suchetha employed a support vector machine (SVM) to classify acetone in breath samples, reaching accuracies of up to 98% [21,22].

Recent developments include DL models like convolutional neural networks (CNN), paired with SVM, achieving up to 98% accuracy in real-time classification [23]. The combination of ML algorithms with dimensionality reduction techniques like singular value decomposition (SVD) and principal component analysis (PCA) has further improved the correlation of diabetes biomarkers [24]. For instance, studies using a voltammetric electronic tongue (VE-tongue) with an e-nose achieved over 99% accuracy in distinguishing diabetic individuals from healthy individuals through PCA and discriminant function analysis (DFA) [25].

Other frameworks have explored lightweight DL models. Zha et al. [26] introduced LTNet, a neural network for classifying VOCs detected by e-noses, achieving 99.06% classification accuracy for lung cancer biomarkers using Gramian Angular Field (GAF) conversion of sensor data into images. Bhaskar et al. [27] developed a hybrid deep neural network model (DNN) for automated T2DM detection, with their correlation neural network (CORNN) achieving 98.02% accuracy in acetone-based DM detection.

In another advancement, Sarno et al. [28] introduced a DNN-based system that integrated e-nose technology for multi-level DM detection. This system utilized discrete wavelet transform (DWT) and PCA for signal preprocessing, achieving 96.29% classification accuracy. Their work demonstrated the superior accuracy of DNNs in classifying BGL levels and distinguishing between healthy, prediabetic, and diabetic individuals.

Ye et al. [29] advanced the field with an e-nose system capable of precise detection and quantitative prediction of BGL through breath analysis. Employing metal oxide (MOX) sensors combined with ML models, particularly Gradient Boosting Tree (GBT), their system achieved 90.4% accuracy in BGL classification with a mean error of 0.69 mmol/L. This study emphasized the potential of e-nose systems as a non-invasive and cost-effective solution for continuous diabetes monitoring. Weng et al. [30] presented a novel integration of e-nose technology within vehicles for real-time diabetes detection. Their system, equipped with 32 MOX sensors, optimized sensor selection using feature extraction and algorithms like Particle Swarm Optimization (PSO) and XGBoost, demonstrating similar effectiveness in BGL detection across populations with T1DM and T2DM.

Kapur et al. [31,32] made notable contributions to real-time diabetes detection through the development of IoT-based systems. Their DiabeticSense system, which employed a Gradient Boosting classification model with multiple sensors to analyze exhaled breath, achieved an accuracy of 86.6%, providing a low-cost, accessible solution for early diabetes detection in rural areas. Building on this, their subsequent innovation, GlucoBreath, further enhanced detection accuracy to 98.4% by leveraging a meta-model that combines Logistic Regression and AdaBoost, offering a portable and user-friendly solution for pre-diagnostic diabetes screening.

Furthermore, the integration of new breath samples into pre-trained algorithms without external computational resources and real-time predictions without cloud-based applications remains an underexplored area. To address the challenges of limited sample sizes and real-time data processing, Gudiño-Ochoa et al. developed a TinyML-powered e-nose system for non-invasive diabetes detection [28]. Their study combines MOX sensors with ML and DL algorithms like XGBoost and DNN for VOC analysis in exhaled breath. This system processes data locally on microcontrollers, enabling real-time, on-device analysis without cloud-based applications [33,34,35,36].

Other studies have explored synthetic data generators for glucose detection [37]. For example, Paleczek et al. validated ML algorithms for acetone detection using an artificial breath system [16,38]. Notably, ML models like XGBoost achieved a precision rate of 99%, while CatBoost produced an absolute error of 0.568 ppm in acetone estimation, underscoring the effectiveness of gradient boosting techniques in managing imbalanced datasets, a common challenge in medical diagnostics. Additionally, they explored the impact of high ethanol concentrations on e-nose response in diabetes detection, demonstrating the importance of considering ethanol levels in non-invasive glucose measurement devices [38].

However, no study to date has reported the experimentation and validation of ML algorithms using synthetic samples generated from real breath tests on a diverse population, including healthy individuals and those with T1DM and T2DM. Moreover, few have addressed the integration of new breath samples into pre-trained algorithms without the need for external computing, utilizing an embedded system with microcontrollers capable of running DL models in real-time. This gap includes applications of e-nose systems for BGL prediction and classification of breath samples between healthy and diabetic individuals [33].

This study aims to generate synthetic data from real exhaled breath samples collected from 58 healthy and diabetic individuals. Using CTGAN, the dataset is expanded to over 14,000 samples, ensuring consistent data distribution [39]. Several algorithms, including gradient boosting machines (XGBoost, LightGBM, and Random Forest), are evaluated for accurate detection without overfitting, based on acetone selectivity in breath samples. Finally, regression and classification methods are implemented in a portable e-nose system with TinyML technology, enabling quantitative and qualitative predictions in a new population using a DNN-compatible model.

This study paves the way for developing a portable e-nose with TinyML technology for real-time DM diagnosis through exhaled breath. By integrating synthetic and real-world data, this work offers a more comprehensive and practical solution for non-invasive diagnostics, advancing the field of diabetes monitoring and laying the groundwork for future innovations in healthcare technologies.

The key contributions of this work include the following:

- Generation of new breath samples using CTGAN, ensuring that ML models are both accurate and generalizable.
- Validation of ML algorithms for the classification of healthy and diabetic individuals and prediction of BGL using synthetic breath data.
- Implementation of a DL model on an embedded TinyML-based e-nose system, enabling real-time, non-invasive diabetes diagnostics.

The paper is organized as follows: Section 1 presents the introduction and literature review, outlining previous work on diabetes prediction using e-noses and exhaled breath analysis; Section 2 describes the system, materials, and methods required for implementation, including details about breath samples collected; Section 3 presents the results; Section 4 discusses the limitations and challenges; and Section 5 summarizes the findings and concludes the study.

## 2. Materials and Methods

The proposed e-nose system was designed to analyze breath samples collected from two distinct participant groups: healthy individuals (HI) and individuals diagnosed with DM, encompassing both T1DM and T2DM. To ensure the accuracy and reliability of the measurements, the system incorporated a range of preprocessing and normalization techniques aimed at reducing sensor noise and enhancing data quality. Data preprocessing played a crucial role in eliminating noise from the patient tests, while advanced methods such as CTGAN were employed to generate synthetic data based on real patient samples. This section provides a comprehensive description of the e-nose setup, detailing the selected gas sensors and microcontroller, as well as the overall system functionality. Additionally, it outlines the calibration procedures specific to the MOX sensors, explains the breath sampling process, and discusses the characteristics of the study participants. The subsequent subsections delve into the methods used for data preprocessing, outlier removal, biomarker correlation analysis, and validation of synthetic data through distribution graphs.

### 2.1. E-Nose Setup

The e-nose system developed for this study builds upon a previously validated design, which has proven its effectiveness in prior research, confirming its suitability for the current investigation. The Supplementary Materials and additional resources can be verified and downloaded from [33]. The system features a sensor array housed within a custom-built cubic acrylic chamber with internal dimensions of 15 cm × 15 cm × 14 cm, providing a total volume of approximately 3.5 L. Breath samples from participants were collected using Tedlar bags with a capacity of 1 L, a method well-documented and widely employed in breath analysis studies [20,24,30,33].

Focusing on the detection of acetone, a key biomarker for T1DM, the e-nose integrates catalytic gas sensors from the MQ series (Waveshare, Shenzhen, China). These sensors were chosen for their ability to detect various compounds, including carbon monoxide, alcohols, ketones, and other VOCs, along with their capability to measure temperature and relative humidity (RH). The selection of these sensors was guided by their successful application in similar research on diabetes detection from exhaled breath, and they were calibrated beforehand to ensure accurate and reliable measurements [14,33]. Table 1 summarizes the sensors used in this device.

**Figure 1:**
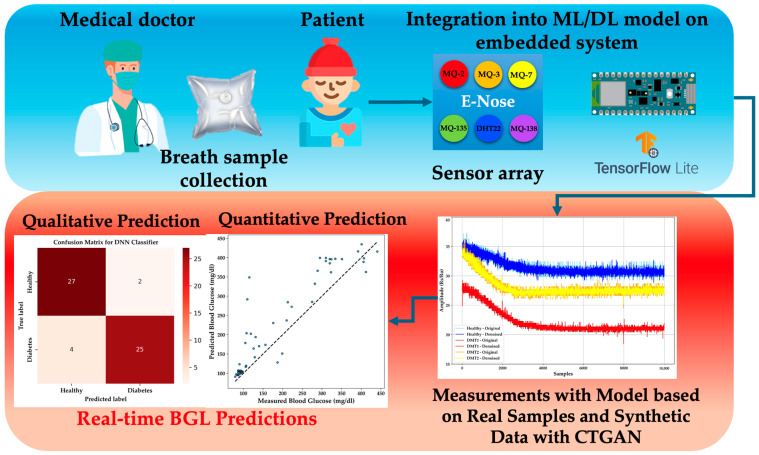
Overview of the non-invasive diabetes detection system using a TinyML-powered e-nose.

The system presented in Figure 1 outlines a non-invasive approach for real-time BGL prediction and DM detection using a TinyML-powered embedded system.

The process begins with the collection of exhaled breath samples from patients, which are then introduced into an e-nose equipped with a sensor array. This array includes MOX sensors specifically designed to detect VOCs such as acetone and alcohol in the breath. Given the sensitivity of MOX sensors to humidity—particularly due to the elevated RH in exhaled breath—a DHT-22 sensor was integrated into the chamber to monitor and regulate humidity levels. High RH can significantly affect sensor accuracy by altering the sensor’s response to VOCs, leading to inconsistent readings or false positives [14].

To mitigate this, a portable dehumidifier was incorporated into the setup. The dehumidifier operates before and after each sampling session to maintain a stable and controlled environment, reducing RH levels within the chamber to acceptable thresholds for the MOX sensors. The MOX sensors were also preheated before each session to improve their sensitivity and performance. While the dehumidifier ensures more accurate readings, it introduces operational complexities, such as the need for regular maintenance and calibration to avoid over-drying the air, which could negatively impact the sensitivity of the sensors. Furthermore, the dehumidifier extends the time required for sample processing, as it must run before each session to ensure optimal sensor performance. Despite these challenges, the dehumidifier remains a crucial component in achieving reliable and reproducible results [13,14,33].

The collected breath samples are analyzed within the sensor chamber, where the sensors detect the presence and concentration of VOCs. The e-nose system processes this data using a pre-trained ML or DL model embedded within the system, implemented via TensorFlow Lite. The model provides both qualitative and quantitative predictions concerning the patient’s BGL. Specifically, the resistive sensors within the e-nose generate Rs/Ro values—where Rs represents the sensor’s resistance in the presence of VOCs and Ro is the baseline resistance in a reference environment. An inverse relationship exists between Rs/Ro values and the concentration of acetone and alcohol; higher Rs/Ro values indicate lower concentrations of these compounds, and vice versa. These readings are crucial for determining BGL and assessing whether the patient is healthy or diabetic [28,33].

To further enhance the system, an Arduino Nano 33 BLE Sense microcontroller (Arduino S.r.l., Monza, Italy) was included, chosen for its compatibility with TinyML and TensorFlow Lite, allowing the direct deployment of ML models on the device [33]. This microcontroller features a 12-bit Analog-to-Digital Converter (ADC) and a 32-bit ARM Cortex-M4 processor, optimizing performance for real-time glucose level detection. Additionally, an LCD screen connected via an I2C communication interface displays real-time data on temperature, humidity, and gas concentrations in parts per million (ppm). Data from breath samples were collected using a serial communication protocol in Python version 3.12, with all recorded measurements stored in CSV format for subsequent analysis. Furthermore, the integration of synthetic data generated by CTGAN enhances the robustness of the model, enabling accurate predictions even with a limited dataset.

### 2.2. Data Pre-Processing

To improve the reliability of the data obtained from the sensor array, a comprehensive preprocessing strategy was employed to minimize signal noise caused by variations in RH, breath temperature, and voltage fluctuations [13,14,20,33,38]. The DWT was applied to the Z-normalized signal to reconstruct the signal and compensate for noise artifacts originating from unstable voltage and variable temperature and humidity conditions [14,28,29]. By decomposing the signal into various frequency components, DWT enables the isolation and removal of noise, ensuring the preservation of the key signal characteristics necessary for accurate sensor readings:

(1) $$DWT\left(f,a,b\right)=\frac{1}{\sqrt{a}}\int_{-\infty }^{\infty }f(t)\psi \left(\frac{t-b}{a}\right)dt$$

The DWT technique allowed the detailed analysis of the signal, enabling the identification of noise components at different scales. By targeting specific frequency bands, noise is effectively filtered while maintaining the integrity of the original signal. This process ensures that critical signal features are retained, which is essential for accurate ML-based predictions. Following the DWT processing, Z-score normalization was applied to the sensor data to standardize the readings across different sensors, ensuring that all variables are on a comparable scale and exhibit properties consistent with a normal distribution [14,28,29,33]. The Z-score normalization is calculated as follows:

(2) $$Z=\frac{X-\mu }{\sigma }$$

where X represents the original sensor reading, μ is the mean of the readings, and σ is the standard deviation. This normalization process is crucial for ensuring that the ML models can effectively handle the data, as it reduces the impact of any outliers and allows for more accurate comparisons between different sensor readings. Following these preprocessing steps, the average values for each sensor’s readings were computed for every participant, resulting in a clean and consistent dataset ready for further analysis.

### 2.3. Breath Sampling and Synthetic Data Generation with CTGAN

**Figure 2:**
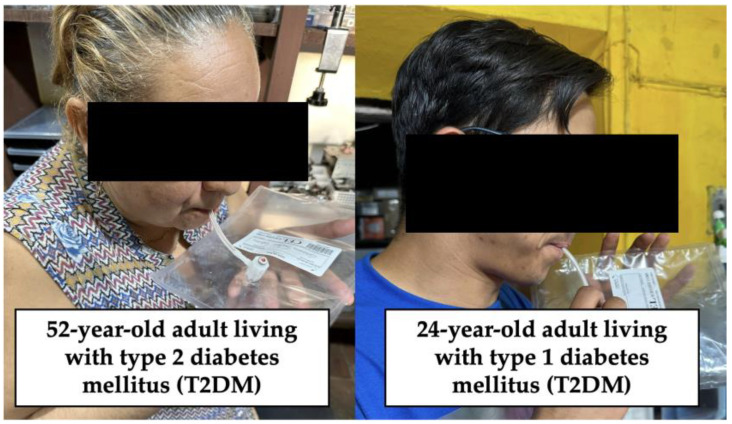
Breath sample collection using Tedlar bags by participants with T1DM and T2DM.

Following sensor calibration, breath samples were collected from 58 participants: 29 healthy individuals, 12 with type T1DM, and 17 with T2DM. Informed consent was obtained from all participants [33]. The e-nose system was preheated for five minutes to stabilize the sensors. Participants were instructed to fully inhale and exhale into a respiratory mouthpiece connected to Tedlar bags, filling them to 90–100% capacity, as depicted in Figure 2. BGL was measured with a glucometer for classification. HI predominantly presented BGL values ranging between 79 and 95 mg/dL, while DM individuals exhibited significantly higher values, with some reaching as high as 300–400 mg/dL, particularly among those not maintaining an adequate quality of life. To capture a wide variability of acetone presence in exhaled breath and its relationship with BGL, samples were collected from the 58 participants at different times: during fasting, after the first meal, after the second meal, and at night [13,14,17,18,19,24,30].

Breath samples were allowed to cool for 5–10 min before being analyzed by the e-nose. The average relative humidity was around 70% and the temperature was approximately 33 °C. During the 90 s measurement period, 10,000 data points were collected per participant using Python and a serial connection. Participant details, including age, gender, and BMI, are provided in Table 2.

The CTGAN method was employed to generate synthetic data that replicates the statistical distributions of the original data. CTGAN is a specialized type of Generative Adversarial Network (GAN) designed to handle tabular data. It consists of two neural networks, the Generator (G) and the Discriminator (D), which are trained in a competitive manner. The goal is for G to learn to generate synthetic data that *D* cannot distinguish from real data [39].

Given a real dataset X, the objective is to train a generator G that can produce synthetic samples such that the distribution of $X_{synthetic}$ is indistinguishable from the distribution of X. Formally, the objective can be expressed as minimizing the loss function of G based on the adversarial cost function, as demonstrated in the equation:

(3) $$\underset{G}{\min}\underset{D}{\max}E_{X∼p_{data}\left(X\right)}\left[\log D\left(X\right)\right]+E_{z∼p_{z}\left(z\right)}\left[\log\left(1-D\left(G\left(z\right)\right)\right)\right]$$

where X represents the distribution of real data, $p_{z}\left(z\right)$ is the distribution of the latent variable z (usually a normal distribution), and $G\left(z\right)$ is the output of the generator given the noise z. For this type of neural network, the generator G learns to map a noise vector z from a latent distribution $p_{z}\left(z\right)$ to the real data distribution $p_{data}\left(X\right)$:

(4) $$G\left(z,c\right)=MLP\left(z\;⨁\;c\right)$$

where c is an added condition (e.g., to handle data categories), z is the noise vector, and ⨁ denotes concatenation. Conversely, the discriminator D, takes a sample X (which can be real or synthetic) and predicts the probability that the sample is real:

(5) $$D\left(X,\;c\right)=\sigma \left(MLP\left(X\;⨁\;c\right)\right)$$

where σ is the sigmoid function that converts the output of the discriminator into a probability between 0 and 1. CTGAN training was conducted over 5000 epochs, using an iterative approach where G and D are alternately updated. This extensive training ensured the generation of synthetic data that encapsulated the inherent variability of the real dataset without overfitting to specific patterns. Additionally, the training enabled the generator to produce high-quality synthetic samples that adhered closely to the underlying statistical properties of the original data. The rounding function was deliberately disabled to avoid distortions in the continuous variables, which can otherwise introduce inaccuracies in predictive models, especially when dealing with fine-grained medical data like BGL. The following parameter update was applied in each iteration:

D Update:

(6) $$\theta_{D}\leftarrow \theta_{D}+\eta \nabla_{\theta_{D}}\left(E_{X∼p_{data}\left(X\right)}\left[\log D\left(X\right)\right]+E_{z∼p_{z}\left(z\right)}\left[\log\left(1-D\left(G\left(z\right)\right)\right)\right]\right)$$

G Update

(7) $$\theta_{G}\leftarrow \theta_{G}-\eta \nabla_{\theta_{G}}E_{z∼p_{z}\left(z\right)}\left[\log\left(1-D\left(G\left(z\right)\right)\right)\right]$$

where $\theta_{D}$ and $\theta_{G}$ are the parameters of the discriminator and generator, respectively, and η is the learning rate. After training, 20,000 new samples $X_{synthetic}$ were generated using the trained generator $G\left(Z\right)$:

(8) $$X_{synthetic}=G\left(Z\right),\;\;\;\;\;z∼p_{z}\left(z\right)$$

In evaluating the quality of the synthetic data generated by the CTGAN model in replicating the distributions of the original variables, the distributions of key variables between the real and synthetic data were compared using estimated density functions, employing Kernel Density Estimation (KDE):

(9) $$\hat{f}(x)=\frac{1}{nh}\sum_{i=1}^{n}K\left(\frac{x-x_{i}}{h}\right)$$

where K is a Kernel function (such as the Gaussian), and h is the bandwidth that controls the smoothness of the estimation.

**Figure 3:**
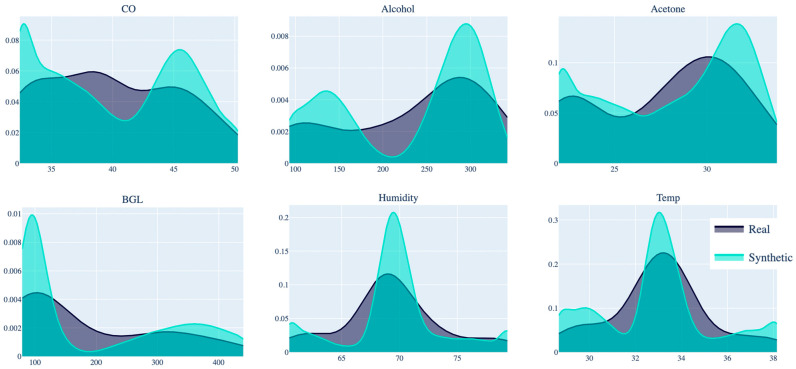
Comparison of real and synthetic data distributions for key variables.

To validate the fidelity of the synthetic data, KDE, a non-parametric method, was applied to compare the distribution of key variables such as acetone, BGL, RH, and temperature between real and synthetic data. As shown in Figure 3, the KDE plots demonstrate that the synthetic data closely replicates the real data’s distribution, particularly in critical variables like acetone and BGL. This validates CTGAN’s ability to faithfully replicate the statistical characteristics of the original data [39]. Specifically, for acetone and CO, there is a close correspondence between the peaks and valleys of the real and synthetic curves, indicating a precise replication of the variability present in the original data. However, slight discrepancies can be observed in some variables, such as RH, which can be attributed to the removal of outliers using the Interquartile Range (IQR) method or to the stochastic nature of the CTGAN model during data generation. The resulting synthetic dataset, after outlier removal, consisted of 14,000 samples. The correlations between acetone and BGL, as detailed in Section 2.3.2, remained consistent and supported the robustness of the model in replicating key clinical relationships.

#### 2.3.1. Removing Outliers

**Figure 4:**
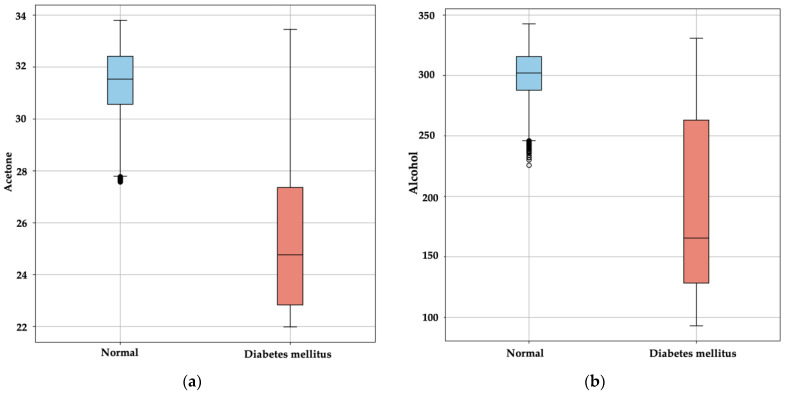
Comparison of biomarker levels in exhaled breath between normal individuals and those with DM: (**a**) Distribution of acetone levels; (**b**) Distribution of alcohol levels.

After generating synthetic data and testing the model, the IQR method was applied to remove outliers. The IQR is calculated as follows:

(10) IQR=Q3−Q1

where *Q*3 represents the third quartile (75th percentile) and *Q*1 represents the first quartile (25th percentile). Outliers are identified as data points that fall below Q1−1.5·IQR or above Q3+1.5·IQR. Figure 4 illustrates the distribution of acetone levels in exhaled breath between normal individuals and those with DM, revealing significant differences between the two groups. Normal individuals exhibit a narrower range of acetone levels, primarily concentrated between 30 and 32 Rs/Ro, with few outliers. In contrast, individuals with DM show a broader range of acetone levels, varying from approximately 24 to 32 Rs/Ro. This suggests that acetone levels can vary more widely in diabetic individuals, and elevated acetone levels could be indicative of the disease, as confirmed in previous studies [17,18,19].

Normal individuals also tend to have higher and less variable alcohol levels in exhaled breath, concentrated within a range of 275 to 325 Rs/Ro. Conversely, individuals with DM exhibit a wider distribution of alcohol levels, ranging from 100 to 300 Rs/Ro. The greater variability in alcohol levels observed in the diabetic group may reflect the complexity of metabolism in these individuals, making this metric a potential indicator for diabetes detection when used alongside other biomarkers like acetone. This variability is crucial, especially when high alcohol concentrations impact the accuracy of diabetes detection through exhaled breath, potentially linked to metabolic alterations in diabetic individuals that result in higher detectable alcohol levels [38].

#### 2.3.2. Biomarkers Correlation and Data Distribution

**Figure 5:**
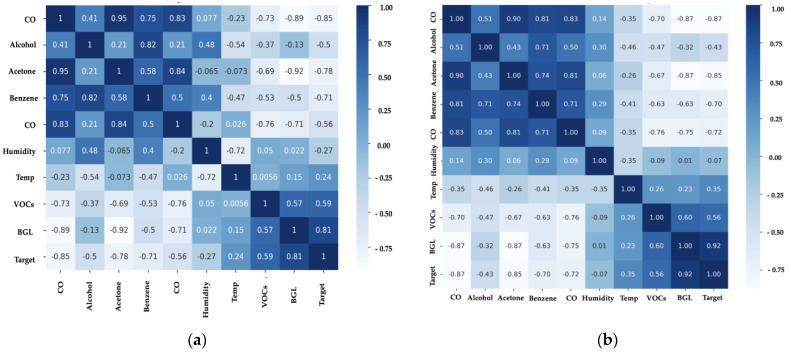
Correlation matrices showing the relationships between various biomarkers in exhaled breath: (**a**) Correlation matrix based on original data, highlighting significant relationships between acetone, BGL, and other variables; (**b**) Correlation matrix based on synthetic data, showing preserved and slightly adjusted correlations after outlier removal and data generation.

In the correlation matrix shown in Figure 5a, significant correlations between various variables can be observed. For instance, acetone shows a strong negative correlation with BGL (−0.92) and the target value (−0.78), indicating that as acetone concentration increases (lower Rs/Ro), BGL tends to be higher, and the likelihood of a DM diagnosis increases. Similarly, CO and alcohol show positive correlations with acetone, which could reflect the interdependence of these compounds in the metabolism of individuals with diabetes. Conversely, temperature shows negative correlations with most chemical variables, which might indicate how environmental conditions affect sensor readings and the concentration of volatile compounds [13,14].

The correlation matrix generated from synthetic data, as shown in Figure 5b, reveals similar patterns but with some notable differences. For example, the correlation between acetone and BGL remains high (−0.87), suggesting that the synthetic data generation model successfully preserved the key relationship between these variables. However, some correlations have slightly shifted, possibly due to the removal of outliers and the inclusion of generated data.

This process of data generation and cleaning appears to have enhanced the consistency of certain relationships between variables, such as the relationship between VOCs and BGL, which shows a slightly higher correlation in the synthetic data (0.60) compared to the original data (0.57). This suggests that including synthetic data may help improve the robustness of the analysis by providing a greater quantity of representative data and reducing the influence of outliers.

**Figure 6:**
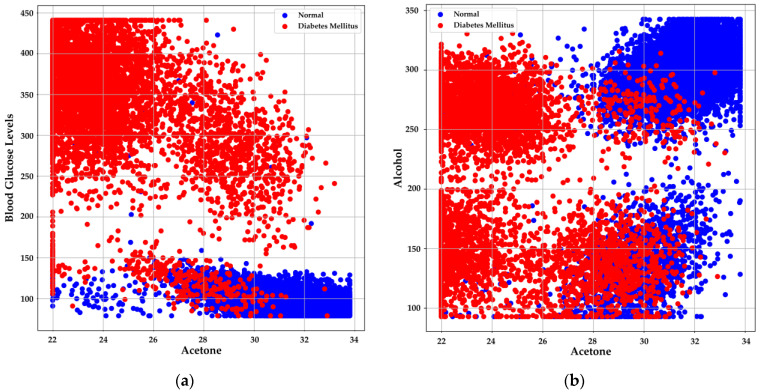
Correlation between Rs/Ro values and biomarkers in exhaled breath. (**a**) Relationship between Rs/Ro values of acetone and BGL in HI and those with DM; (**b**) Relationship between Rs/Ro values of acetone and alcohol levels, highlighting the differences between HI and those with DM.

Normal individuals tend to exhibit higher Rs/Ro values for both acetone and alcohol, indicating lower concentrations of these compounds in exhaled breath. Conversely, individuals with DM (depicted in red) display lower Rs/Ro values, suggesting higher concentrations of acetone and alcohol. This pattern, as shown in Figure 6, implies that the concentrations of these compounds could be instrumental in distinguishing between normal and diabetic individuals [16,28,33,38].

The correlation between acetone Rs/Ro values and BGL concentration was analyzed to evaluate the sensors’ predictive capability. The results demonstrate that individuals with DM have lower Rs/Ro values for acetone and higher BGL, while normal individuals exhibit higher Rs/Ro values for acetone and lower BGL [28,33]. This finding, illustrated in Figure 6a, suggests that acetone Rs/Ro values could serve as a reliable indicator of BGL concentration, supporting their use in non-invasive diabetes monitoring systems. Figure 6b illustrates that normal individuals tend to cluster in regions with higher Rs/Ro values for both acetone and alcohol, signifying lower concentrations of these compounds. In contrast, individuals with DM are more widely distributed across lower Rs/Ro values, corresponding to higher concentrations of acetone and alcohol. This distribution further supports the potential use of these biomarkers in distinguishing between normal and diabetic individuals [13,14,29].

## 3. Results

This section presents the outcomes of the study, focusing on the performance evaluation of various classification and regression models for predicting DM and BGL concentration, alongside their compatibility with TinyML for real-time implementation. The results include a comparative analysis of ML models such as XGBoost, Random Forest, LightGBM, and DNN, with a focus on their predictive accuracy, computational efficiency, and suitability for deployment in embedded systems. Additionally, the integration of the selected model into an Arduino Nano BLE Sense microcontroller is discussed, highlighting its application in non-invasive diabetes monitoring.

### 3.1. Classification Models for Qualitative Prediction of DM

The dataset was stratified into training and testing subsets, employing an 80–20 split to ensure that the models were evaluated on unseen data. A standard scaler was utilized to normalize the features, a crucial step for maintaining numerical stability and ensuring that all input features contributed equally to the model’s learning process. A comprehensive GridSearch was conducted to fine-tune the hyperparameters for each classification algorithm, optimizing for performance metrics such as accuracy, precision, recall, and F1-score [16]. Each of these metrics provides a different perspective on how well the models are performing in distinguishing between HI and those with DM. Here are the equations for these metrics:

(11) $$Accuracy=\frac{TP+TN}{TP+TN+FP+FN}$$

(12) $$Precision=\frac{TP}{TP+FP}$$

(13) $$Recall=\frac{TP}{TP+FN}$$

(14) $$F1-Score=2\cdot \frac{Precision\;\cdot Recall}{Precision+Recall}$$

where TP are True Positives (correctly predicted positives), TN are True Negatives (correctly predicted negatives), FP are False Positives (incorrectly predicted positives), and FN are False Negatives (incorrectly predicted negatives). Recall, also known as sensitivity or true positive rate, measures the proportion of actual positives that are correctly identified by the model. The F1-Score is especially useful when there is an uneven class distribution, as it balances the need to avoid false positives and false negatives.

**Figure 7:**
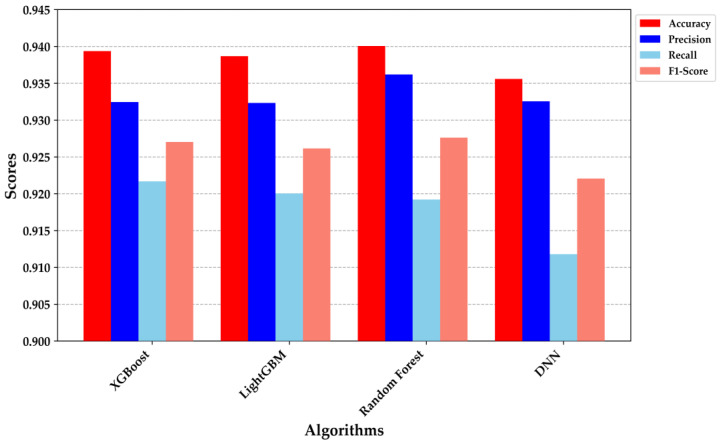
Comparative performance metrics (accuracy, precision, recall, F1-score) for XGBoost, LightGBM, Random Forest, and DNN models.

Figure 7 provides a detailed comparison of the performance metrics across the four classification models: XGBoost, LightGBM, Random Forest, and DNN. XGBoost and Random Forest demonstrated superior performance, each achieving an accuracy of approximately 0.94. The precision and recall metrics were particularly noteworthy; XGBoost exhibited a precision of 0.935 and a recall of 0.920, while Random Forest achieved a precision of 0.930 and a recall of 0.925. These results indicate that both models are highly effective at correctly identifying true positives while minimizing false negatives, making them robust for clinical decision-making scenarios.

The DNN model, despite having a slightly lower accuracy (0.930), demonstrated a well-balanced precision and recall, indicating its strong generalization capability and suitability for deployment in resource-constrained environments, such as embedded systems. The performance metrics underscore the trade-offs among different algorithms, with each excelling in specific areas. For example, while the DNN was marginally outperformed in overall accuracy, its balanced F1-score suggests it could be more reliable in situations where both false positives and false negatives are equally critical. These findings affirm the appropriateness of these models for non-invasive diabetes classification, with XGBoost and Random Forest standing out due to their superior precision and recall. The integration of such models into a TinyML framework holds promise for providing real-time, on-device decision support, thereby enhancing the scalability and accessibility of diabetes diagnostics.

**Figure 8:**
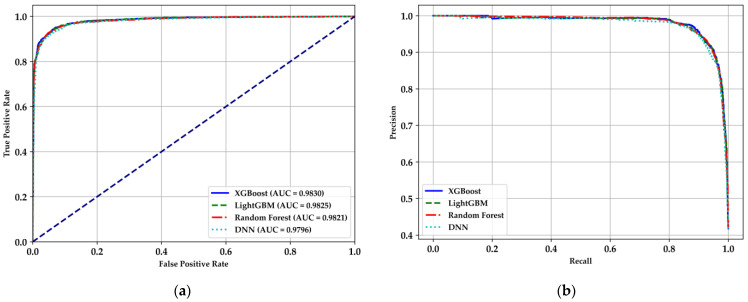
ROC and Precision–Recall curves for the classification models: (**a**) ROC curves demonstrating the discriminative ability of XGBoost, LightGBM, Random Forest, and DNN models; (**b**) Precision–Recall curves highlighting the balance between precision and recall across the models.

The analysis of the receiver operating characteristic (ROC) and Precision–Recall curves in Figure 8 provides valuable insights into the performance of the classification models. In Figure 8a, the ROC curves for XGBoost, LightGBM, Random Forest, and DNN models are depicted. All models demonstrate a high area under the ROC curve (AUC), with values exceeding 0.97, indicating excellent discriminative ability in classifying between healthy individuals and those with DM. The XGBoost model exhibits the highest AUC (0.9829), closely followed by LightGBM (0.9825), Random Forest (0.9810), and DNN (0.9794). These results suggest that all models are highly effective, with XGBoost showing a slight edge in terms of overall classification performance.

Figure 8b presents the Precision–Recall curves, which are particularly informative in the context of imbalanced datasets where the proportion of positive (diabetic) cases is lower. The curves illustrate that all models maintain high precision across a wide range of recall values, reflecting their ability to minimize false positives while correctly identifying a significant portion of true positives. The DNN, despite slightly lower precision at high recall levels, shows robust performance comparable to the other models. The close overlap of the curves further emphasizes the comparable effectiveness of the models, though XGBoost and LightGBM slightly outperform in balancing precision and recall. Overall, these curves reinforce the suitability of these models for the qualitative prediction of DM, with XGBoost and LightGBM standing out as particularly reliable choices for classification tasks in this context [16,27,29,32,33,38].

**Figure 9:**
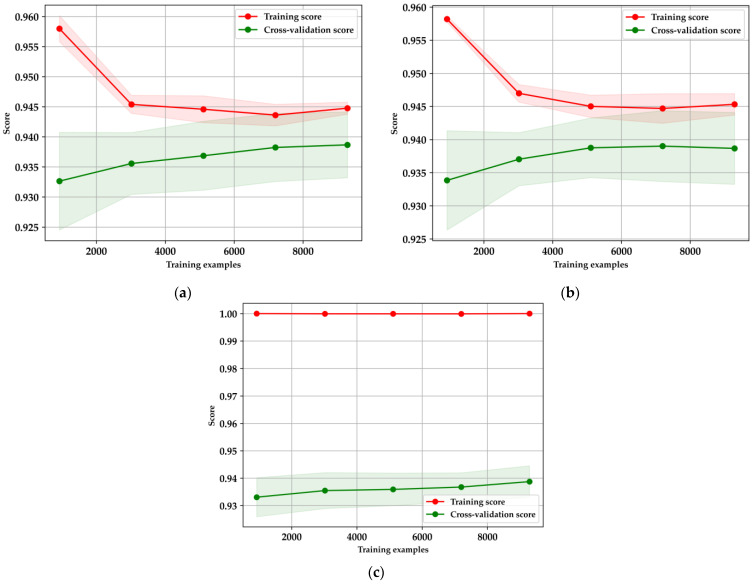
Learning curves for classification models: (**a**) XGBoost; (**b**) LightGBM; (**c**) Random Forest.

The learning curves in Figure 9 illustrate the performance and generalization capabilities of the XGBoost, LightGBM, and Random Forest models, each evaluated using five-fold cross-validation. For XGBoost (Figure 9a), there is a noticeable decline in the training score as the number of training examples increases, indicating reduced overfitting and a shift towards more generalized learning. Simultaneously, the cross-validation score gradually rises and stabilizes, suggesting that the model’s performance improves with more data, ultimately reaching a point of convergence. Similarly, LightGBM (Figure 9b) follows this pattern, where the training score decreases as more data is introduced, reflecting a positive reduction in overfitting. The cross-validation score for LightGBM also shows improvement, albeit with some variance, before stabilizing, demonstrating its effectiveness in leveraging larger datasets [16,38,40]. In contrast, the Random Forest model (Figure 9c) maintains a consistently high training score, which may indicate overfitting, particularly with smaller datasets. Despite this, the cross-validation score gradually improves as more data is introduced, underscoring the model’s need for careful tuning and sufficient data to generalize effectively.

**Figure 10:**
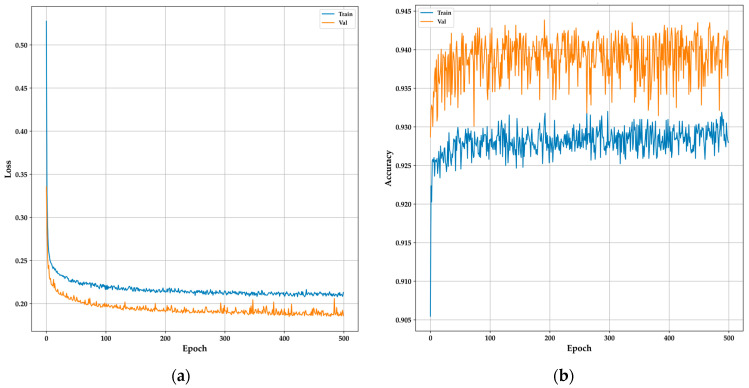
Training and validation curves for the DNN model: (**a**) Loss curves showing the stabilization of training and validation loss over 500 epochs; (**b**) Accuracy curves demonstrating consistent performance with higher validation accuracy compared to training accuracy.

The training and validation curves for the DNN model provide a clear overview of its learning behavior and generalization capabilities over 500 epochs. The model architecture comprised an input layer with 20 units, followed by a hidden layer containing 1200 units, both employing ReLU activation functions and L1 regularization to mitigate overfitting. Additionally, the Adamax optimizer was utilized to enhance performance. Dropout layers with a 0.1 rate were added after each dense layer to further enhance generalization. The output layer consisted of a single unit with a sigmoid activation function for binary classification. In Figure 10a, the loss curve shows a sharp initial decrease in both training and validation losses, indicating that the model is quickly learning from the data. As training progresses, the losses stabilize, with the validation loss consistently lower than the training loss, suggesting strong generalization without overfitting. Figure 10b, depicting the accuracy curves, reveals that the training accuracy gradually improves, stabilizing around 93%, while the validation accuracy consistently remains higher, around 94%. This consistent performance across both curves indicates that the DNN model effectively captures the essential features for accurate classification, making it a reliable candidate for real-time, non-invasive diabetes monitoring in resource-constrained environments like TinyML-powered e-nose systems, as demonstrated in similar studies with DNN [28,33].

**Figure 11:**
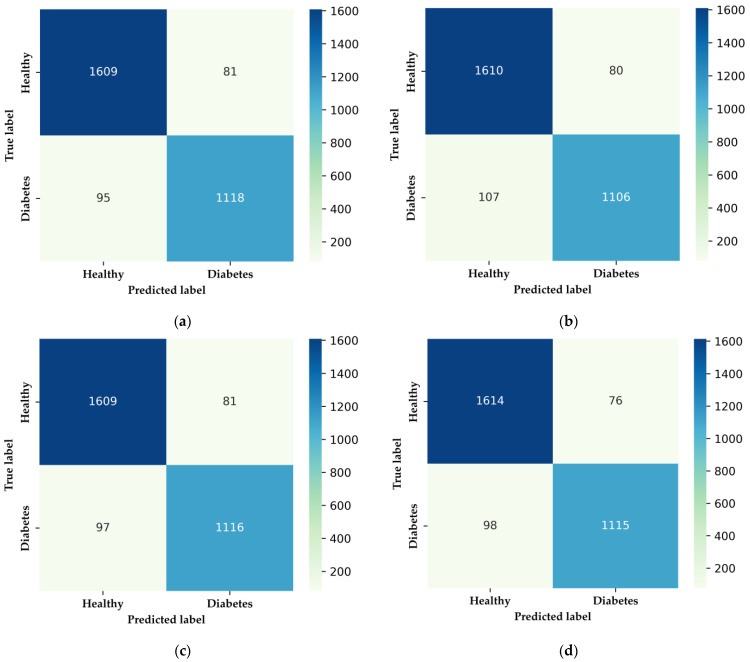
Confusion matrices for the classification models: (**a**) XGBoost model showing high true positive and true negative rates; (**b**) DNN model illustrating balanced classification performance; (**c**) LightGBM model with a strong distinction between healthy and diabetic cases; (**d**) Random Forest model demonstrating high accuracy in classification.

The confusion matrices in Figure 11 illustrate the classification performance of four ML models—XGBoost, DNN, LightGBM, and Random Forest—used to distinguish between healthy individuals and those with DM. XGBoost (Figure 11a) achieves strong accuracy with 1609 true positives and 1118 true negatives, but it shows 81 false positives and 95 false negatives, indicating room for improvement in minimizing false negatives. The DNN model (Figure 11b) demonstrates a balanced classification with 1610 true positives and 1106 true negatives but has slightly more false negatives (107), which suggests it might struggle with borderline cases. LightGBM (Figure 11c) performs similarly to XGBoost, with 1609 true positives and 1116 true negatives, along with 81 false positives and 97 false negatives, showing robustness in classification. Random Forest (Figure 11d) exhibits the highest true positive rate with 1614 true positives and 1115 true negatives, and the lowest false positives (76), making it particularly effective in correctly identifying healthy individuals while maintaining a relatively low false negative rate. Overall, while all models demonstrate high accuracy, Random Forest and XGBoost slightly outperform the others in minimizing false negatives, which is critical in medical diagnostics where missed diagnoses can have serious implications.

### 3.2. Regression Models for Prediction of BGL Concentration

**Figure 12:**
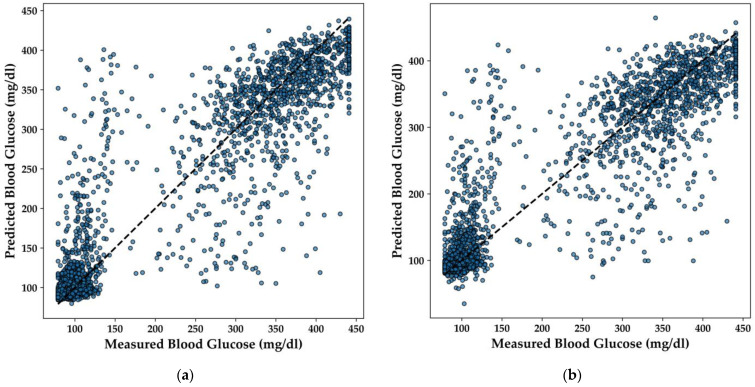

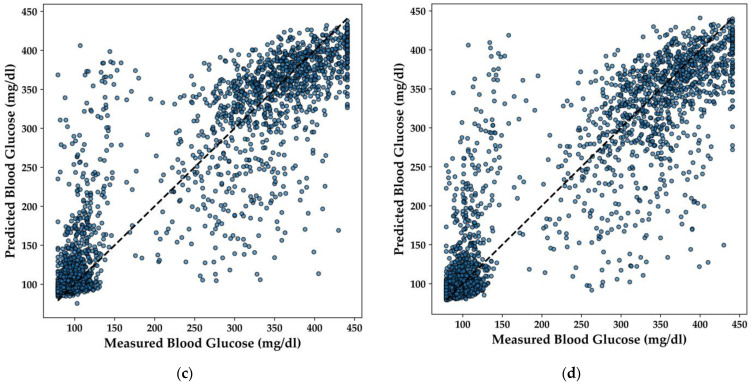
Scatter plots comparing predicted and measured BGL for regression models: (**a**) LightGBM; (**b**) Extra Trees; (**c**) XGBoost; (**d**) DNN.

In this section, the performance of various regression models for predicting BGL is analyzed, with a particular focus on the LightGBM, Extra Trees, XGBoost, and DNN models. The scatter plots in Figure 12 illustrate the correlation between the predicted and measured BGL concentrations for each model.

The LightGBM model (Figure 12a) demonstrates a strong linear relationship between predicted and actual BGL values, with predictions closely clustering around the line of perfect agreement. This indicates that the model effectively captures the underlying patterns in the data, yielding accurate predictions across a range of BGL values.

Similarly, the Extra Trees model (Figure 12b) shows a high degree of accuracy, with the majority of points aligning well with the diagonal line. The distribution of points suggests that the model handles both high and low BGL values effectively, offering balanced performance across the dataset. The XGBoost model (Figure 12c) also exhibits a strong predictive capacity, with points tightly clustered around the line of perfect agreement. This model’s robustness is evident, as it maintains high accuracy even in areas where the data is more dispersed, ensuring reliable predictions for BGL concentrations.

Lastly, the DNN model (Figure 12d) demonstrates consistent performance, with predicted values closely matching the actual measurements. The scatter plot indicates that the model effectively generalizes across different BGL levels, making it a suitable candidate for integration into real-time, non-invasive diabetes monitoring systems.

Overall, these regression models exhibit strong predictive performance, with each model offering distinct advantages that could be leveraged depending on the specific requirements of the application. The DNN model, in particular, stands out for its capacity to generalize across the data, a characteristic essential for deployment in resource-constrained environments like TinyML-powered e-nose systems.

To complement the analysis of the regression models for predicting BGL, a detailed comparison of the models in terms of their R^2^ score, Mean Absolute Error (MAE), and training time is provided. As shown in Table 3, the LightGBM model outperformed the others with an R^2^ score of 0.86 and the lowest MAE of 28.35 mg/dL, while also having the fastest training time of only 0.12 s. In contrast, the DNN took significantly longer to train—623.41 s—while achieving an R^2^ score of 0.84 and an MAE of 30.13 mg/dL. The Extra Trees and XGBoost models both performed similarly with R^2^ scores of 0.84, but the Extra Trees model exhibited a slightly higher MAE of 30.23 mg/dL compared to XGBoost’s 29.82 mg/dL. This detailed comparison highlights the trade-offs between model accuracy, error, and computational efficiency, emphasizing the strengths of LightGBM in this application.

In all evaluated models, acetone consistently emerges as the most critical feature, with a significantly higher importance score than other biomarkers such as benzene, CO, alcohol, and various VOCs.

**Figure 13:**
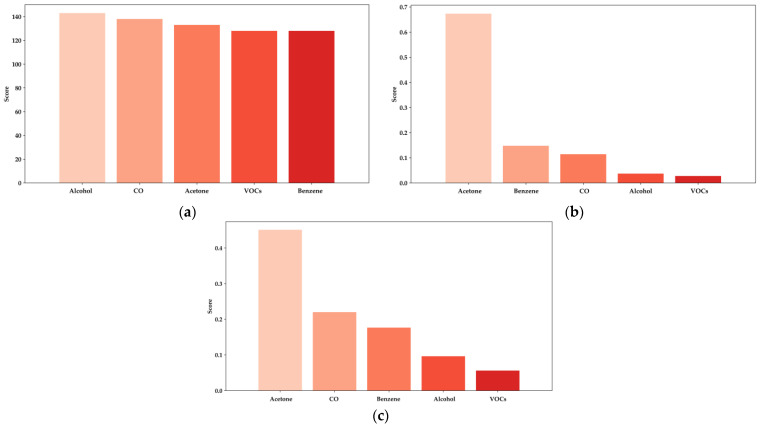
Feature importance analysis for predicting BGL: (**a**) XGBoost; (**b**) LightGBM; (**c**) Random Forest.

Figure 13a, representing the XGBoost model, highlights acetone’s dominant role in predicting BGL, contributing significantly more than other features [14,16,38]. Similarly, in Figure 13b, the LightGBM model reflects this pattern, where acetone’s contribution surpasses that of any other biomarker, particularly emphasizing its importance in non-invasive glucose monitoring. Notably, alcohol also shows considerable relevance in the LightGBM model, confirming its impact on predictions, as previously established in studies focused on the influence of ethanol concentration in breath analysis for DM detection. This further supports the necessity of incorporating alcohol measurements in e-nose systems to enhance the accuracy of BGL predictions and diabetes diagnostics [38].

In the Random Forest model, shown in Figure 13c, acetone remains the most influential feature, although other biomarkers like benzene and CO also show slightly increased importance compared to the XGBoost and LightGBM models. This variation suggests that while acetone is the primary biomarker, the contribution of additional features like alcohol and other VOCs can further enhance the predictive power of these models, especially in diverse and resource-constrained environments.

The analysis summarized in Table 4 quantifies these importance scores, reinforcing the robust performance of the models in leveraging the most relevant biomarkers for accurate BGL prediction. The insights provided by this analysis are crucial for optimizing e-nose systems and refining the ML algorithms used in non-invasive diabetes detection, thereby enhancing their effectiveness and reliability in real-world healthcare applications.

### 3.3. TinyML with TensorFlow Lite Application with DM and HI Patients

In this section, the application of TinyML with TensorFlow Lite for the qualitative classification of DM and HI, as well as the regression of BGL, is discussed. The DNN model was selected for these tasks due to its compatibility with the TensorFlow ecosystem, which allows for seamless conversion to TensorFlow Lite and deployment on resource-constrained devices like microcontrollers [33,34,35,36].

**Figure 14:**
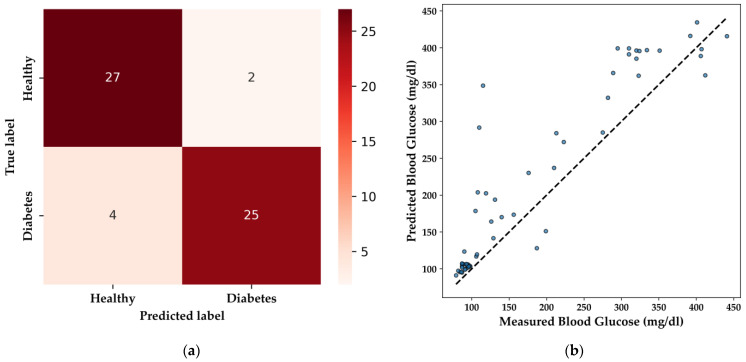
Performance evaluation of the DNN model in TinyML: (**a**) Confusion matrix for binary classification of DM and HI patients; (**b**) Scatter plot showing predicted vs. measured BGL.

The DNN model’s performance in qualitative classification is illustrated by the confusion matrix (Figure 14a). The model correctly identifies 27 healthy patients and 25 patients with diabetes, with minimal misclassifications, including two false positives and four false negatives. This level of performance is particularly important in medical applications, where the accurate identification of diabetic individuals is essential to avoid potential misdiagnosis, ensuring that patients receive the appropriate care.

For BGL regression, the DNN model’s predictive capability is demonstrated in the scatter plot (Figure 14b), with an R^2^ score of 0.736 and an MAE of 41.00 mg/dL. These metrics indicate a reasonably strong correlation between predicted and actual BGL values, making the model suitable for non-invasive glucose monitoring in embedded systems. Notably, the DNN model effectively predicted BGL levels within the normal range (85–100 mg/dL) for the HI group, and while predictions for elevated BGL levels (around 200 mg/dL) were accurate, there remains a need for a broader distribution of high BGL samples to further refine the model’s performance.

**Figure 15:**
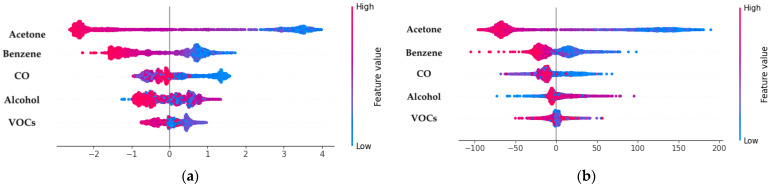
SHAP analysis for DNN model interpretability: (**a**) SHAP summary plot for classification of DM and HI patients; (**b**) SHAP summary plot for regression of BGL.

To enhance the interpretability of the classification and regression models, we applied SHAP values to explain the contributions of individual features to the predictions [41]. The SHAP summary plots (Figure 15a,b) illustrate the impact of various VOCs on the model’s predictions for both classification and regression tasks. In Figure 15a, the SHAP summary plot for classification reveals that acetone is the most influential feature, with higher concentrations indicating a greater likelihood of DM. The distribution of SHAP values also highlights the impact of other VOCs, such as benzene and CO, which contribute to the model’s decision-making process.

Figure 15b presents the SHAP summary plot for regression, where acetone again emerges as the dominant feature influencing BGL predictions. The positive correlation shown in the plot suggests that increases in acetone levels correspond to higher predicted BGL values, affirming its role as a key biomarker for DM.

The decision to exclusively test the DNN model stems from the specific challenges associated with converting other ML models, such as XGBoost, LightGBM, and Random Forest, into formats compatible with TensorFlow Lite. These models, while powerful and effective in various predictive tasks, often require complex conversion processes [33]. These processes can lead to variations in performance metrics due to differences in numerical precision and implementation between the original algorithms and TensorFlow. Additionally, the lack of direct support for these models in TensorFlow’s conversion tools posed significant barriers to their integration into a microcontroller-based system. Given these constraints, the DNN model was chosen for its ease of integration, maintaining robust performance and meeting the critical requirements for real-time diabetes monitoring in a resource-limited environment.

The DNN model was tested under various conditions, including fasting, postprandial (after the second meal of the day), and nighttime, across the same cohort of 58 patients. For each of these conditions, the e-nose system with the integrated DNN model was used separately, ensuring that the model’s performance could be reliably assessed in diverse real-life scenarios. Additionally, it is essential to consider the habits and variability among DM patients, as factors such as stress, anxiety, menstrual cycles for women, and physical exercise significantly influence daily glucose levels. These variables were considered during the testing phases to ensure that the DNN model could generalize well across different patient profiles and day-to-day conditions [40].

The DNN model implemented within a TinyML framework using TensorFlow Lite demonstrates high effectiveness in the binary classification of DM and HI patients, as well as in the regression of BGL. The model’s robust performance, coupled with its seamless integration into resource-constrained environments, makes it an ideal candidate for non-invasive, real-time monitoring devices [33,34,35,36]. This study demonstrates that using CTGAN to validate and improve models with a diverse patient population and their exhaled breath analysis provides a solid foundation for future work aimed at enhancing the model’s accuracy and applicability in broader healthcare applications, while also addressing the challenges associated with deploying other ML models in similar environments.

## 4. Discussion

The integration of TinyML with e-nose technology for detecting DM through exhaled breath has demonstrated considerable potential, aligning with recent advances in non-invasive diagnostic techniques. This study aimed to enhance the effectiveness of ML models, particularly in resource-constrained environments, by utilizing synthetic data generated through CTGAN. The models validated in this research, including LightGBM and Random Forest, showed strong predictive capabilities for BGL and diabetes detection, with the inclusion of synthetic data significantly improving their robustness [16,30,33,38].

Despite these promising results, several limitations and challenges were identified. One primary concern is the sensitivity of the MOX sensors used in the e-nose to high RH levels [13,14,27,38]. These sensors require continuous validation and calibration to maintain accuracy, particularly given their susceptibility to environmental factors. The implementation of a dehumidifier in the e-nose setup, while effective, introduces additional complexity in sensor maintenance and operational logistics.

Additionally, while CTGAN proved effective in expanding the dataset to over 14,000 synthetic samples, it may introduce certain limitations, particularly regarding the diversity of real-world cases. The synthetic data generated may not fully capture the variability present in real breath samples, which could limit the generalizability of the model’s predictions. Specifically, in this study, BGL ranges predominantly fell between 80–120 mg/dL and above 180 mg/dL, with extreme cases reaching up to 400 mg/dL in individuals with poorly managed diabetes. However, broader testing across a more diverse range of BGL is necessary to improve model accuracy. Moreover, multiple factors can influence breath analysis for BGL prediction, namely, medical histories and lifestyles, including carbohydrate intake, physical activity, and insulin administration (particularly for T1DM), which are not easily captured in synthetic data [41].

To improve model reliability and ensure more comprehensive generalization, future studies should include a broader and more diverse participant pool. Target groups should encompass a wide range of ages, ethnic backgrounds, and varying degrees of diabetes severity, including prediabetic individuals and those with comorbidities such as cardiovascular disease. Moreover, it would be beneficial to incorporate additional biomarkers beyond acetone and ethanol, such as ketones, lactate, and inflammatory markers, which can provide more nuanced insights into metabolic states and disease progression. Expanding the participant pool and incorporating a more diverse set of biomarkers will provide a more comprehensive representation of real-world variability, thereby enhancing the robustness and generalizability of the model [13,14,38,41].

Furthermore, the presence of ethanol in exhaled breath, as highlighted in [38], can affect BGL predictions and model accuracy. Ethanol levels may vary significantly throughout the day depending on the individual’s diet or alcohol consumption, further complicating the analysis. The recognition of ethanol’s role underscores the need for careful consideration of multiple VOCs when developing non-invasive glucose monitoring systems. A standardized protocol for breath sample collection that accounts for these variations (e.g., fasting, postprandial, or post-exercise states) is essential to minimize variability and ensure accurate predictions. Establishing a rigorous breath sampling protocol across different patient groups (HI and DM) would greatly enhance the generalization and performance of the integrated e-nose system in real-time BGL predictions and classification tasks [24,28,30].

The reliance on DL models, particularly DNN, for embedded system implementation presents both strengths and limitations. While the DNN model demonstrated robust performance in both qualitative classification and BGL prediction tasks, the conversion of other ML models like XGBoost, LightGBM, and Random Forest into formats compatible with TensorFlow Lite posed significant challenges. These models often require complex conversion processes, which can lead to variations in performance metrics due to differences in numerical precision and implementation between the original algorithms and TensorFlow. This limitation highlights the need for more compatible ML libraries and frameworks for TinyML integration [33,34,35]. To contextualize the performance of the models used in this study, Table 5 compares the best classifier models from recent studies on diabetes detection, while Table 6 highlights the leading regressor models for BGL prediction.

To address the challenges associated with deploying models on TinyML frameworks, future research should explore lightweight DL models that do not compromise the efficiency of the device. This approach aims to balance the computational and memory constraints of microcontrollers while maintaining high performance in real-time diabetes monitoring applications. Exploring and developing such models could significantly enhance the applicability and scalability of TinyML-powered e-nose systems in broader healthcare contexts [33].

Moreover, the study’s exclusive focus on breath analysis for diabetes detection, while innovative, should be expanded to include other diagnostic parameters, such as vital signs (blood pressure, heart rate, SpO_2_). Integrating these additional features could enhance the interpretability and accuracy of the models, providing a more comprehensive diagnostic tool. Previous studies have demonstrated the benefits of combining breath analysis with other physiological data for more accurate diabetes detection and BGL prediction [31,32].

While this study has demonstrated the feasibility and effectiveness of using TinyML-powered e-nose systems for non-invasive diabetes monitoring, several challenges remain. Future research should focus on overcoming the limitations related to sensor sensitivity, environmental factors, and model compatibility with TinyML. Additionally, expanding the scope of diagnostic parameters and increasing the sample size will be crucial in refining these systems for broader healthcare applications. The integration of alcohol (ethanol) as a key feature in models like LightGBM, along with the continued exploration of synthetic data generation techniques, offers a solid foundation for enhancing the accuracy and applicability of these systems in real-world medical settings [38].

## 5. Conclusions

This study has demonstrated the potential of integrating ML models with e-noses for non-invasive diabetes monitoring. By addressing the limitations of small sample sizes using CTGAN, the research successfully generated over 14,000 synthetic samples from real-world tests. This approach enhanced the robustness and accuracy of the models used for diabetes detection and BGL prediction, achieving high accuracy rates of 86% for BGL identification with LightGBM and 94.14% for diabetes detection with Random Forest.

The implementation of TinyML in e-nose systems proved effective for real-time healthcare applications. However, challenges were encountered in deploying certain ML models due to compatibility issues with TinyML. The decision to focus on the DNN model was due to its seamless integration with TensorFlow Lite, enabling deployment on resource-constrained devices [33,35].

Future research should explore lightweight DL models that maintain device efficiency and consider alternative ML models such as CatBoost and NGBoost. Additionally, it is crucial to implement a more rigorous protocol for breath sample collection, particularly for diabetic patients whose conditions can vary widely due to factors like diet, physical activity, and insulin use. Incorporating features such as vital signs could enhance the interpretability and accuracy of the models, making the e-nose a more reliable tool for both qualitative classification and quantitative prediction of BGL [16,23,24,25,27,28,29,30,31,32,33].

While the study marks significant progress in non-invasive diabetes monitoring, ongoing research is needed to refine these systems. Expanding datasets, exploring new models, and integrating additional predictive features will further enhance the utility of this technology as a diagnostic tool, providing fast, reliable, and painless diabetes detection [13,14].

## 6. Patents

A Utility Model application has been submitted to the Mexican Institute of Industrial Property (IMPI). This application has successfully passed the formal examination, complying with the requirements established by the Federal Law on Industrial Property and the Regulations of the Industrial Property Law in Mexico. The Utility Model has been published in the IMPI database, SIGA 2.0, as of 15 February 2024, under application number MX/u/2023/000465. The authors associated with this patent are Alberto Gudiño-Ochoa, Julio Alberto García-Rodríguez, Jorge Ivan Cuevas-Chávez, Raquel Ochoa-Ornelas, and Daniel Alejandro Sánchez-Arias.

## Figures and Tables

**Table 1:** Sensors used in E-nose system developed [33].

Sensor	Target Gases	Detection Range of Target Gas	Environment Condition Working
MQ-2	$H_{2}$$,\;\mathrm{LPG},\;{\mathrm{CH}}_{4}$, CO, Alcohol, Propane, Air	200–10,000 ppm CO	Temperature: −10–50 °C RH: less than 95% Standard detecting condition: 20 ± 2 °C temperature, 65 ± 5% humidity
MQ-3	$\mathrm{Alcohol},\;\mathrm{Benzine},\;{\mathrm{CH}}_{4}$, Hexane, LGP, CO, Air	0.1–10 mg/L Alcohol	
MQ-7	$H_{2}$$,\;\mathrm{CO},\;\mathrm{LPG},\;{\mathrm{CH}}_{4}$, Alcohol, Air	50–4000 ppm CO	
MQ-135	${\mathrm{CO}}_{2}$$,\;\mathrm{Alcohol},\;\mathrm{Air},\;{\mathrm{NH}}_{4}$, Toluene, Acetone, CO	0–200 ppm Acetone	
MQ-138	$\mathrm{Benzene},\;\mathrm{CO},\;{\mathrm{CH}}_{4}$, n-Hexane, Alcohol, Propane, Air	200–10,000 ppm Benzene	
DHT-22	Temperature, Relative Humidity	−40–80 °C Temperature, 0–100% Relative Humidity	Temperature: 0–50 °C RH: 0–100%
MICS-5524	$\mathrm{CO},\;\mathrm{VOCs},\;C_{2}H_{6}$ $\mathrm{OH},\;H_{2}$ $,\;NH_{3}$ $,\;{\mathrm{CH}}_{4}$	1–1000 ppm VOCs	Temperature: 23 ± 5 °C RH: less than 95%

**Table 2:** Physical information of 58 participants.

**Parameter**	**Healthy Patients**	**Diabetes Mellitus Patients**
Age (yr.)	25.71 ± 2.01	30.67 ± 5.81
Height (cm)	1.71 ± 0.58	1.70 ± 0.22
Weight (kg)	76.11 ± 9.22	77.91 ± 13.51
BMI (kg/m^2^)	26.47 ± 4.89	28.97 ± 7.81
Gender (M/F)	15 M/14 F	13 M/16 F
Number of participants by health individuals (HI) or Type of diabetes mellitus (T1DMI/T2DMI)	29 HI	12 T1DMI/17 T2DMI
Minimum and maximum BGL (mg/dL)	79.81/98.91	121.48/407.82

**Table 3:** Performance metrics and training time for regression models in quantitative prediction BGL.

Regressor	Train Time (s)	R^2^ Score	Mean Absolute Error
LightGBM	0.12	0.86	28.35
XGBoost	1.11	0.84	29.82
DNN	623.41	0.84	30.13
Extra Trees	0.23	0.84	30.23

**Table 4:** Feature importance scores for acetone, benzene, CO, alcohol, and VOCs across different regression models used for BGL prediction.

Model	Acetone	Benzene	CO	Alcohol	VOCs
LightGBM	134.57	129.71	139.71	144.86	129.71
XGBoost	0.66	0.15	0.11	0.4	0.3
DNN	0.3555	0.0214	0.0053	0.0086	0.0116
Extra Trees	0.45	0.17	0.22	0.1	0.06

**Table 5:** Comparison of best classifier models from recent studies on DM detection using E-nose data.

Study	Best Classifier Model	Dataset Type (Real or Artificial)	Year	Accuracy (%)	Precision (%)	Recall (%)	F1-Scores (%)
Lekha S. et al. [23]	1D-CNN with SVM	Real: 26 individuals	2018	98	98	99	98
Paleczek A. et al. [16]	XGBoost	Artificial breath simulations	2021	99	97.9	100	97.4
Weng X. et al. [30]	Random Forest	Real: 240 individuals	2023	93.33	97.05	89.9	92.8
Zaim O. et al. [25]	SVM-DFA	Real: 60 individuals	2023	93.75	-	-	-
Bhaskar N. et al. [27]	CORNN with SVM	Real: 152 individuals	2023	98	97	98.5	97.8
Gudiño-Ochoa A. et al. [33]	XGBoost	Real: 44 individuals	2024	95	95	95	95
Kapur R. et al. [32]	GBoost-XGBoost	Real: 492 individuals	2024	95.8	96.9	-	96.1
Present study	Random Forest	Artificial: 14,000 samples (from 58 individuals)	2024	94	93	92.5	91

**Table 6:** Comparison of Best Regressor Models for BGL Prediction.

Study	Best Model Regressor	Dataset Type (Real or Artificial)	Year	R^2^ Score
Ye Z. et al. [29]	GBR	Real: 41 individuals	2023	0.873
Kapur R. et al. [32]	LR-AdaBoost	Real: 492 individuals	2024	0.984
Present Study	LightGBM	Artificial: 14,000 samples (from 58 individuals)	2024	0.86

## Data Availability

The original data presented in this study are available upon request from the corresponding author due to ethical and privacy considerations. The synthetic data generated during this study, which replicates the statistical characteristics of the original data, are openly available at https://github.com/AlbertoGudinoOchoa/breath-diabetes-synthetic-data/ under the MIT License (accessed on 24 September 2024).

## Supplementary Materials

The following supporting information can be downloaded at: www.mdpi.com/1424-8220/24/4/1294 (accessed on 24 September 2024).
